# Association between serum and adipose tissue resistin with dysglycemia in South Asian women

**DOI:** 10.1038/s41387-019-0071-3

**Published:** 2019-02-18

**Authors:** Sulochana Wijetunge, R. M. C. J. Ratnayake, H. M. S. R. B. Kotakadeniya, Shanthini Rosairo, Kembra Albracht-Schulte, Latha Ramalingam, Naima Moustaid-Moussa, Nishan Sudheera Kalupahana

**Affiliations:** 1 Department of Pathology, Faculty of Medicine, University of Peradeniya, Peradeniya, Sri Lanka; 2 Department of Obstetrics and Gynecology, Faculty of Medicine, University of Peradeniya, Peradeniya, Sri Lanka; 3 Department of Surgery, Faculty of Medicine, University of Peradeniya, Peradeniya, Sri Lanka; 4 Department of Radiology, Faculty of Medicine, University of Peradeniya, Peradeniya, Sri Lanka; 5 Department of Physiology, Faculty of Medicine, University of Peradeniya, Peradeniya, Sri Lanka; 6 Department of Nutritional Sciences and Obesity Research Cluster, Texas Tech University, Lubbock, TX USA

## Abstract

**Background/Objectives:**

Mechanisms of obesity-associated insulin resistance and dysglycemia in South Asians remain relatively unknown. The objective of this study was to detect subcutaneous (SAT) vs. visceral (VAT) adipose tissue characteristics and adipocytokines associated with obesity, insulin resistance, and dysglycemia in South Asian women.

**Subjects/Methods:**

This was a hospital-based cross-sectional study conducted in Sri Lanka. Subjects comprised of 58 adult women who underwent routine abdominal surgeries. SAT and VAT were obtained from anterior abdominal wall and omentum, respectively. Measures of adiposity, serum insulin and glucose, SAT and VAT crown-like structures (CLS), macrophages, resistin by immunohistochemistry, mean adipocyte area (MAA), and serum adipocytokines were examined.

**Results:**

The homeostatic model assessment for insulin resistance (HOMA-IR) score significantly correlated with age and waist circumference (WC), but not with body mass index (BMI). Although the number of CLS positively correlated with BMI, there were no significant differences between the number of CLS in women with normal fasting glucose (NFG) vs. those with impaired fasting glucose (IFG), indicating that adipose tissue macrophage infiltration is unlikely to be related to dysglycemia. In contrast, serum resistin level was on average 60% higher in women with IFG compared to ones with NFG (*p* < 0.05). Serum resistin levels correlated with age (*r* = 0.36, *p* < 0.05) and WC (*r* = 0.27, *p* < 0.05). There were no associations in serum levels of other adipocytokines with IFG. Adipose immunohistochemistry showed that women with IFG had a higher percentage of resistin positive adipocytes in SAT compared to ones with NFG. MAA of VAT, but not SAT, correlated with both BMI and WC.

**Conclusions:**

Resistin may be an important adipokine linking central adiposity and insulin resistance in South Asian women. Both systemic and adipose tissue resistin are linked to dysglycemia in these individuals and may be a potential biomarker for diabetes in this population.

## Introduction

Type-2 diabetes mellitus (T2DM) is a major health problem in the world as well as in South Asia^1^. A combination of insulin resistance and pancreatic beta cell dysfunction are mechanistically responsible for the pathogenesis of T2DM^2^. Obesity, especially visceral adiposity, is a major risk factor for insulin resistance and T2DM^3^. Obese individuals develop insulin resistance, which is attributed to (A) a chronic low-grade inflammation in white adipose tissue, (B) dysregulation of adipocytokine secretion, and (C) ectopic lipid deposition and lipotoxicity^4^. Adipose tissue inflammation can lead to dysregulation of adipocytokine secretion that can induce insulin resistance in insulin-sensitive tissues, such as skeletal muscle and liver.

While mechanisms responsible for dysglycemia and insulin resistance have been studied in detail in western populations, these mechanisms have hitherto been unexplored in detail in South Asians^5^. Indeed, individuals of South Asian descent have a higher risk of developing T2DM for a given body mass index (BMI) compared to Caucasians. Therefore, it is likely that different mechanisms operate in the pathogenesis of obesity-associated insulin resistance and dysglycemia in South Asians^5^. It is especially important to dissect these mechanisms given the recent escalation of obesity^6^ and its co-morbidities in this region^7^.

Our aim in the current study was to understand the mechanisms leading to insulin resistance and dysglycemia in South Asian women. We hypothesized that defective adipose tissue expansion during positive energy balance would give rise to dysregulated adipocytokine secretion, which in turn leads to insulin resistance and dysglycemia in South Asian adults. To test this hypothesis, we studied metabolic markers, adipocytokines, and visceral (VAT), and subcutaneous white adipose tissue (SAT) characteristics of adult women who were hospitalized for routine abdominal surgery.

## Materials/subjects and methods

### Subjects

The study group comprised 58 adult women who presented at Teaching Hospital, Peradeniya, Sri Lanka for a routine abdominal surgery (Age range 18–75; hysterectomy, *n* = 23; laparoscopy, *n* = 9; explorative laparotomy, *n* = 6; para-umbilical hernia repair, *n* = 6; other, *n* = 14). The Ethical Review Committee of the Faculty of Medicine, University of Peradeniya, approved this study. Patients with T2DM, inflammatory conditions, cancer, or previous abdominal surgery were excluded. Demographic data, diet, and physical activity history were obtained using an interviewer-administered questionnaire. Height was measured by a stadiometer to the nearest millimeter. Weight was measured by a digital scale to the nearest 100 g. Waist circumference was measured midway between the lowest rib and the superior border of the iliac crest in the mid axillary line, with an inelastic measuring tape at the end of normal expiration to the nearest millimeter. BMI was calculated [BMI = weight (kg)/height^2^ (m^2^)]. BMI cutoffs for Asians were used to classify different BMI categories.

### Serum biomarkers and adipocytokines

Following an overnight fast, blood was collected into eppendorf tubes and allowed to clot on ice. Serum was separated via centrifugation. The supernatant was frozen and stored for subsequent analysis. Serum glucose was measured by the glucose oxidase method. Serum insulin (EMD Millipore, Billerica, MA, USA, Cat. #EZHI-14K), high-molecular weight (HMW) adiponectin (EMD Millipore, St. Charles, Missouri, USA, Cat. #SPREZHMWADPN65K), Interleukin (IL)−8 (Bio-Techne Corporation, Minneapolis, MN, USA, Cat. #D8000C), IL-10 (RayBiotech, Inc., Norcross, GA, USA, Cat. #ELH-IL-10), chemerin (BioVendor, Asheville, NC, USA, Cat. #RD191136200R), omentin-1 (BioVendor, Asheville, NC, USA, CAT. #RD191100200R), and high-sensitivity C-reactive protein (hsCRP) (MP Biomedicals, Solon, OH, USA; Cat. # 07BC-1119) were assayed using commercially available ELISA kits. Serum tumor necrosis factor (TNF)-alpha, total adiponectin and resistin were measured utilizing Luminex XMAP technology Magpix™, Human Adipokine Magnetic Bead Panel I (Millipore, Billerica, MA, USA; Cat. # HADK1MAG-61K). Milliplex® Human Adipokine Magnetic Bead Panel II (EMD Millipore, Billerica, MA, USA; Cat. # HADK2MAG-61K) was used to measure IL-6, leptin, and monocyte chemotactic protein (MCP)-1. Homeostatic model assessment for insulin resistance (HOMA-IR) score was calculated as fasting glucose (mmol/L) x fasting insulin (mU/L) / 22.5.

### Adipose tissue histology and immunohistochemistry

During surgery, samples of greater omentum (VAT) and fat from the anterior abdominal wall midway between the umbilicus and the pubic symphysis (SAT) were collected and fixed with formalin. Paraffin embedded and hematoxylin and eosin (H&E) stained tissue sections were prepared. The number of crown-like structures (CLS) per 25 fields of x10 objective (Olympus CX 31microscope) were calculated by a histopathologist blinded to the patient details. Adipocyte size was measured using the ImageJ software (mean surface area of 100 adipocytes in each biopsy). Immunohistochemistry (IHC) for resistin and CD 68 was performed on paraffin embedded sections using heat induced antigen retrieval method. The primary antibodies used were polyclonal rabbit anti resistin antibody (LS Bio, LS-B12981) and monoclonal mouse CD 68 (Dako, clone PG-M1, Code–Nr.M 0876). For visualization, a secondary antibody kit with peroxidase detection system was used (Dako REAL EnVision, detection system, Peroxidase/DAB, rabbit/mouse). CD 68 was used to confirm the presence of macrophages in adipose tissue sections with or without forming CLS. Resistin expression in adipocytes was assessed and granular cytoplasmic positivity was regarded as true positivity. The amount of resistin positive adipocytes in a given tissue section was expressed as a percentage of all adipocytes by visual assessment. The strength and number of resistin positivity in macrophages when present in tissue sections were also documented.

### Ultrasound scan of the anterior abdominal wall

The thickness of anterior abdominal wall was measured in five places (left and right upper, left and right lower and midline around umbilicus) by ultrasound scanning and the recoding was obtained in centimeters. Similar techniques have been employed previously to estimate the subcutaneous fat content in the abdominal wall^8^.

### Data analysis

Sample size was determined by a priori power calculation, based on estimated detectable differences and standard deviations in adipocyte size and HOMA-IR score sufficient to achieve 80% statistical power for the detection of a difference with *p* < 0.05. Data were analyzed using Microsoft Excel (2013), SPSS (Statistical and Products Service Solutions, Chicago) version 20 and GraphPad Prism software. Pearson’s correlations were calculated to examine relations between variables. Mean values of continuous variables between two groups were compared using the independent samples *t*-test. One-way ANOVA followed by post hoc testing was used to compare more than two groups.

## Results

### Subject characteristics

The mean age of women in the study group was 46.1 years (SD = 12.7) with a mean BMI of 25.4 (SD = 5.9). Of the 58 women, 18 (31%) were overweight (BMI between 23 and 27.49) and 19 (33%) were obese (BMI ≥ 27.5) as per the south Asian BMI scale^9^, while 43 women (74%) had abdominal obesity (waist circumference ≥ 80 cm).

### Variables associated with insulin resistance and dysglycemia

**Fig. 1:**
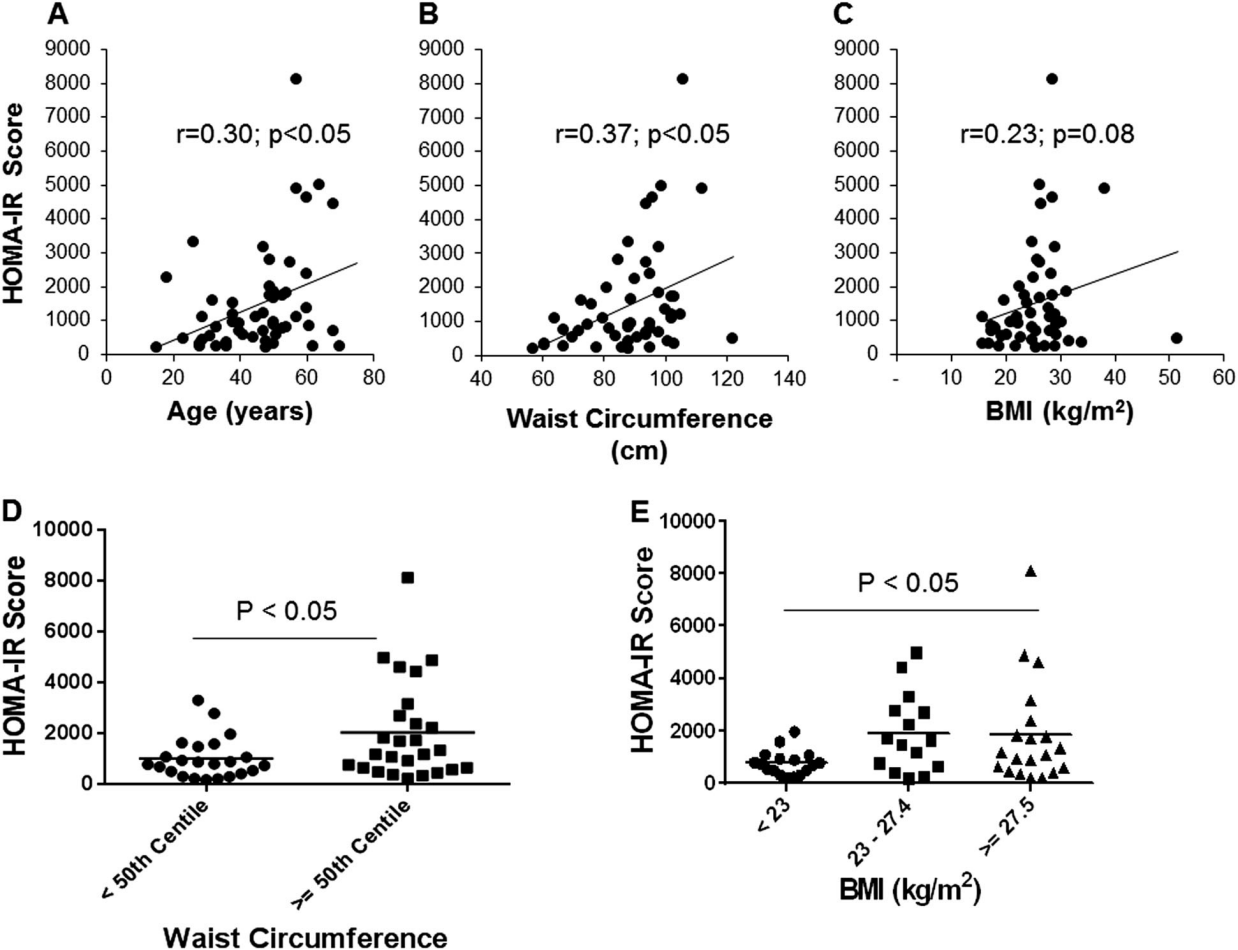
Variables associated with insulin resistance. Correlations between HOMA-IR score and age (**a**), waist circumference (**b**), and BMI (**c**) are shown. HOMA-IR scores of women whose waist circumference was in the lower 50^th^ vs. the score in women in the upper 50^th^ centile (**d**) and in different BMI categories (**e**) are shown. *N* = 58. BMI: body mass index, HOMA-IR: homeostatic model assessment for insulin resistance

First, we calculated the HOMA-IR score to assess the degree of insulin resistance in these women. As illustrated in Fig. 1, HOMA-IR score exhibited significant positive correlations with age and waist circumference. Although the correlation between the HOMA-IR score and BMI was not significant, obese (BMI ≥ 27.5) women had a significantly higher HOMA-IR score compared to normal weight women (BMI < 23). Women having a higher waist circumference (≥50^th^ centile in the group) also had a significantly higher HOMA-IR score compared to ones in the lower 50^th^ centile, indicating that insulin resistance develops with increasing adiposity, especially in the abdominal compartment.

**Fig. 2:**
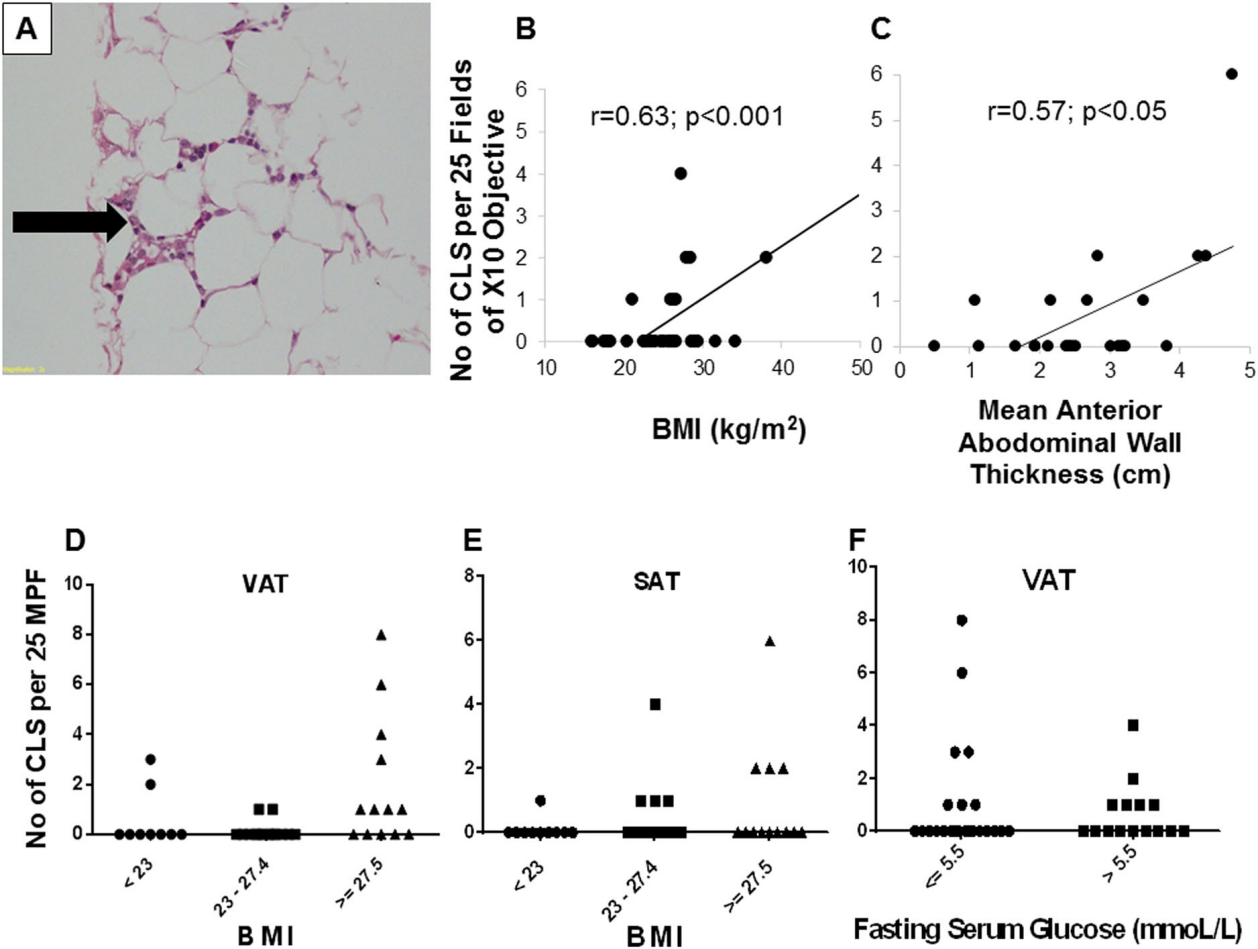
Adipose tissue crown-like structures. Adipose tissue crown-like structures (CLS-arrow in **a**), correlation of number of CLS per 25 fields of x10 objective with BMI (**b**) and mean anterior abdominal wall thickness (**c**) are shown. Number of CLS between different BMI categories in VAT (**d**) and SAT (**e**), and between women with normal fasting glucose vs. impaired fasting glucose (**f**) are also shown. *N* = 36 for mean anterior abdominal wall thickness; *n* = 38 for CLS and 58 for others. BMI: body mass index, VAT: visceral adipose tissue, SAT: subcutaneous adipose tissue

Next, we investigated potential mechanisms associated with insulin resistance and increasing visceral adiposity. A chronic low-grade inflammation characterized by immune cell infiltration in the adipose tissue is causally linked to systemic insulin resistance^10^. These adipose tissue immune cell aggregates, which comprise macrophages and other immune cell types such as T cells and B cells, can be identified as crown-like structures (CLS) under light microscopy^11,12^. Therefore, we next analyzed H&E stained sections of VAT and SAT for CLS (*n* = 38). As expected, the number of CLS per 25 fields of x10 objective positively correlated with BMI (Fig. 2).

To better understand the association between increased adiposity and adipose tissue inflammation, we measured the thickness of the anterior abdominal wall (AAWT) by ultrasound scanning in a subset of women (*n* = 36) and found that this thickness positively correlated with the number of CLS (*r* = 0.57; *p* < 0.05). Although the aforementioned associations provided evidence for adipose tissue inflammation, only 33% of women had one or more CLS. Moreover, CLS were mainly found at higher levels of BMI (Fig. 2d, e). Finally, the number of CLS was not different between women with normal fasting glucose (NFG) vs. impaired fasting glucose (IFG) (Fig. 2f). Therefore, adipose tissue macrophage infiltration per se is unlikely to be the major mechanism responsible for dysglycemia in these South Asian women.

### Characteristics of women with normal fasting glucose vs. those with impaired fasting glucose

White adipose tissue is an active endocrine organ. Adipose tissue hormones (adipocytokines) can be dysregulated in obesity, which can lead to insulin resistance and dysglycemia^10^. Thus, we next analyzed the serum levels of major adipocytokines including leptin, adiponectin, resistin, TNF-alpha, IL-6, IL-8, IL-10, omentin-1, and chemerin, as well as the systemic inflammatory marker hsCRP in women with NFG vs. those with IFG (Table 1). As expected, women with IFG had a higher waist circumference and HOMA-IR score compared to women with NFG. Of the studied adipocytokines, serum resistin was on average about 60% higher in the women with IFG (*p* < 0.05).

**Table 1:** Subject characteristics and serum adipokines between women with normal fasting glucose (NFG) and those with impaired fasting glucose (IFG)

Variable	NFG (*n* = 38)		IFG (*n* = 20)		*p*-value
	Mean	(SD)	Mean	(SD)	
Age (years)	43.0	(13.4)	52.9	(8.4)	0.006*
Weight (kg)	57.8	(16.3)	62.5	(8.8)	0.262
BMI (kg/m^2^)	24.6	(6.4)	27.0	(4.5)	0.170
WC (cm)	85.7	(13.7)	95.2	(8.4)	0.009*
HC (cm)	92.4	(11.8)	96.6	(10.5)	0.207
FSG (mmoL/L)	4.8	(0.5)	6.3	(0.5)	<0.001*
Serum insulin (uU/L)	5.4	(5.3)	7.3	(7.4)	0.291
HOMA-IR score	996.4	(1098.5)	2141.8	(2095.7)	0.009*
AAWT (cm)	3.0	(1.2)	3.9	(0.9)	0.052
VAT-MAA (uM^2^)	6817.8	(3140.5)	7710.2	(2433.9)	0.378
SAT MAA (uM^2^)	9135.1	(5319.8)	9134.1	(1878.9)	0.999
Serum hsCRP (mg/L)	3.2	(5.2)	3.6	(5.4)	0.776
Serum adiponectin (Total-µg/mL)	24.7	(85.7)	10.1	(6.4)	0.473
Serum adiponectin (HMW-µg/mL)	7.5	(7.7)	7.3	(7.9)	0.922
Serum resistin (ng/mL)	35.6	(23.8)	58.7	(49.8)	0.022*
Serum leptin (ng/mL)	216.2	(463.1)	110.8	(238.9)	0.415
Serum TNF-alpha (pg/mL)	165.1	(72.7)	181.4	(54.8)	0.650
Serum MCP-1 (pg/mL)	20.0	(44.5)	20.4	(29.0)	0.972
Serum omentin-1 (ng/mL)	354.1	(156.3)	391.4	(184.9)	0.440
Serum chemerin (ng/mL)	206.8	(46.7)	205.4	(47.1)	0.915
Serum IL-6 (pg/mL)	7.8	(11.7)	5.8	(6.3)	0.528
Serum IL-8 (pg/mL)	32.0	(42.6)	51.2	(91.5)	0.319
Serum IL-10 (pg/mL)	14.2	(17.5)	10.6	(16.1)	0.634

*BMI:* body mass index, *WC:* waist circumference, *HC:* hip circumference, *FSG:* fasting serum glucose, *HOMA:* Homeostatic model assessment, *IR:* insulin resistance, *AAWT:* anterior abdominal wall thickness, *VAT:* visceral adipose tissue, *SAT:* subcutaneous adipose tissue, *MAA:* mean adipocyte area, *hsCRP:* high-sensitivity C-reactive protein, *TNF:* tumor necrosis factor, *MCP:* monocyte chemotactic protein, *IL:* interleukin

*p  < 0.05

### Serum and adipose tissue resistin

**Fig. 3:**
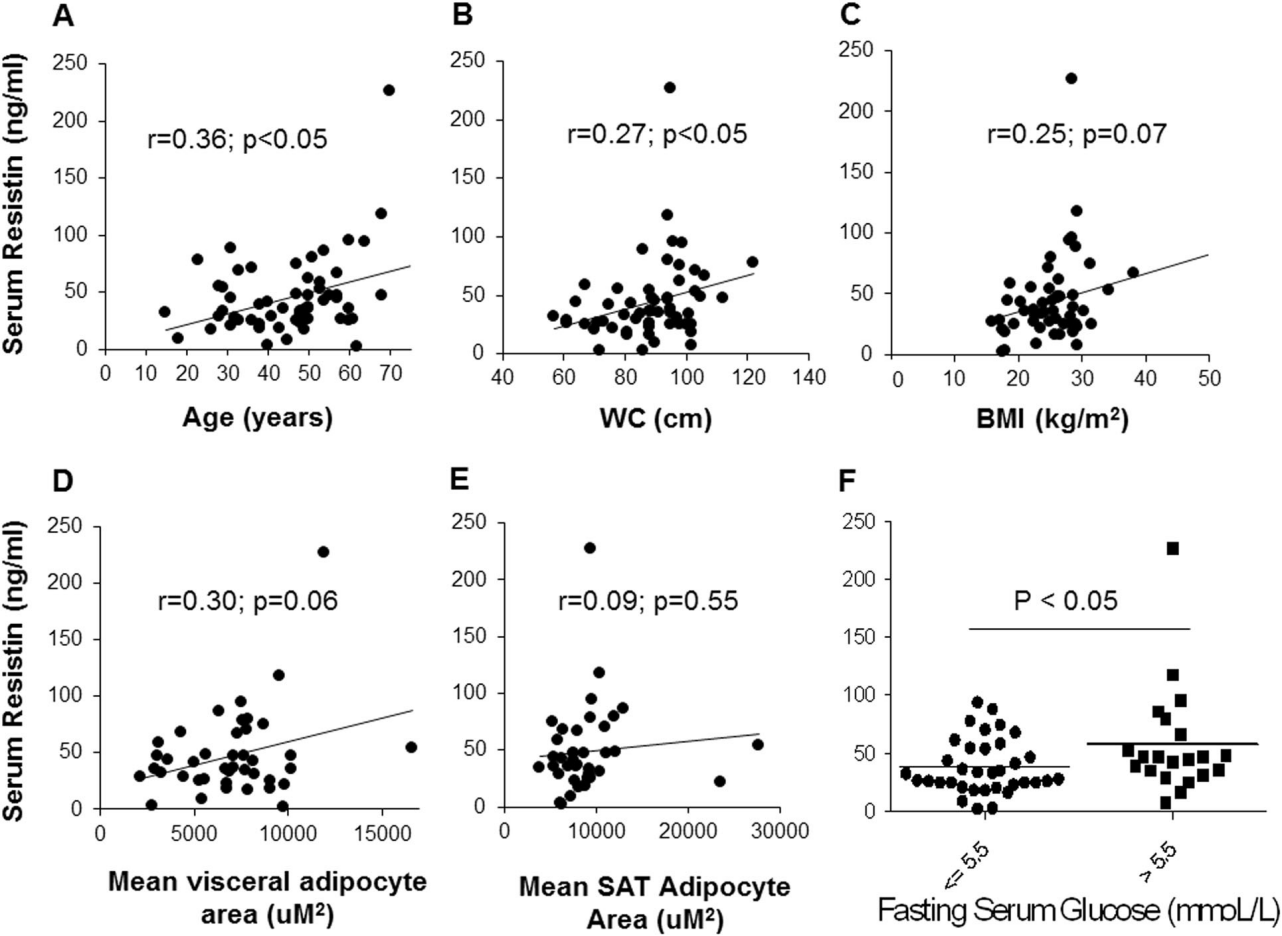
Factors associated with serum resistin levels. Correlations between serum resistin and age (**a**), waist circumference (**b**), BMI (**c**), mean VAT adipocyte area (**d**), and mean SAT adipocyte area (**e**) are shown. Serum resistin levels were compared between women with normal fasting glucose vs. impaired fasting glucose (**f**). *N* = 40 for adipocyte area and 58 for others. BMI: body mass index, VAT: visceral adipose tissue, SAT: subcutaneous adipose tissue

Since resistin is known to be associated with insulin resistance^13^ and because we found that women with IFG have higher resistin levels (Table 1), we next investigated factors associated with serum resistin. Figure 3 shows that serum resistin increases with age and waist circumference, and was trending significance with increasing BMI. This potentially indicated that this adipokine is a potential link between increased visceral adiposity and dysglycemia. Since adipocyte size is linked to adipocyte function and adipocytokine secretion^14^, next we investigated the relation between adipocyte size and resistin levels. While there was a trend for a positive correlation between mean VAT adipocyte area and serum resistin level, this was not significant. There was no association between SAT adipocyte area and resistin (Fig. 3d, e). Finally, there was a positive correlation between serum resistin and IL-6 levels (*r* = 0.337, *p* = 0.029), while none of the other studied adipocytokines correlated with resistin.

**Fig. 4:**
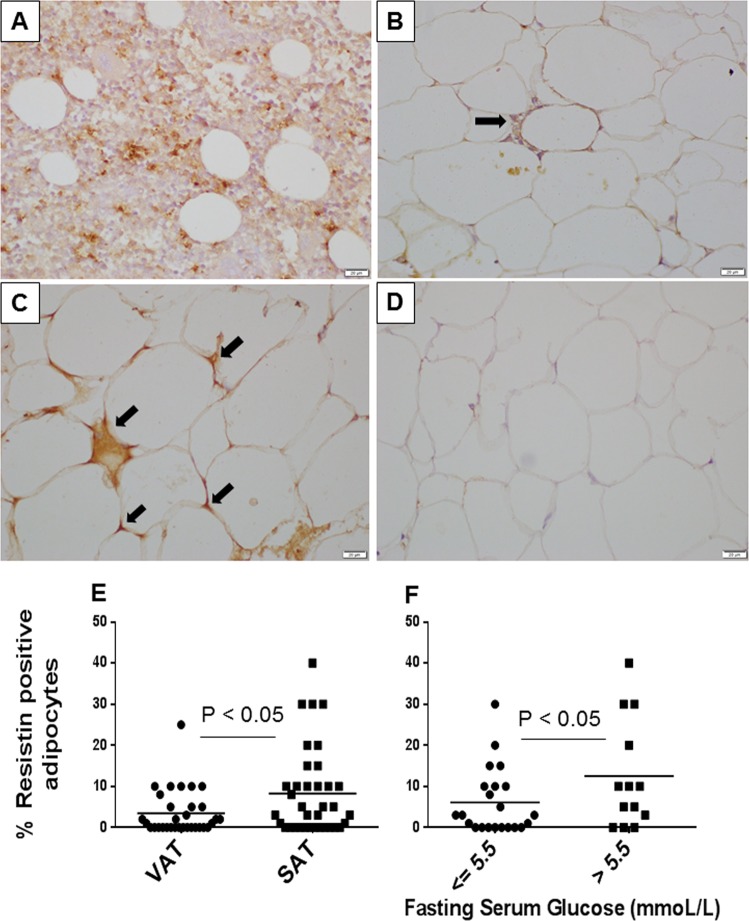
Resistin immunostaining. Immunostaining of bone marrow tissue as a positive control for resistin (brown stain) was performed (**a**). Next, resistin immunostaining of adipose tissue was performed and representative resistin positive adipose tissue macrophages (**b**), resistin positive (**c**), and negative (**d**) adipocytes are shown. Resistin expression in adipocytes was assessed and granular cytoplasmic positivity was regarded as true positivity. The amount of resistin positive adipocytes in a given tissue section was expressed as a percentage of all adipocytes by visual assessment, which was compared between VAT and SAT (**e**) and in women with normal fasting glucose vs. impaired fasting glucose (**f**). *N* = 36. VAT: visceral adipose tissue, SAT: subcutaneous adipose tissue

It is well understood that resistin is primarily secreted by adipocytes in rodents; however, the source of resistin in humans is controversial. Therefore, we performed immunohistochemistry of adipose tissue to study the resistin levels in this tissue. We found that some, but not all adipocytes secrete resistin (Fig. 4). Moreover, adipocytes stained stronger for resistin than macrophages and the overall macrophage infiltration in adipose tissue was sparse, suggesting that adipocytes may be the primary source of resistin in the adipose tissue in these women. SAT had a higher percentage of resistin positive adipocytes compared to VAT, indicating depot-specific differences in resistin secretion (Fig. 4d). Importantly, women with IFG had a significantly higher percentage of resistin positive adipocytes in the SAT compared with participants with NFG (Fig. 4e), suggesting that local resistin secretion in adipose tissue may be linked to dysglycemia. However, there was no correlation between serum resistin level and the percentage of resistin positive cells in adipose tissue, suggesting that adipocyte resistin may not be the only contributor to serum resistin.

### Adiposity, adipocyte size, and insulin resistance

Adipose tissue expansion in obesity occurs via adipocyte hyperplasia and hypertrophy^15^. The latter is characterized by increased adipocyte size and is associated with insulin resistance^15^. To gain an understanding about the adipocyte expansion in these women, we compared the adipocyte area of different depots with adiposity measures.

**Fig. 5:**
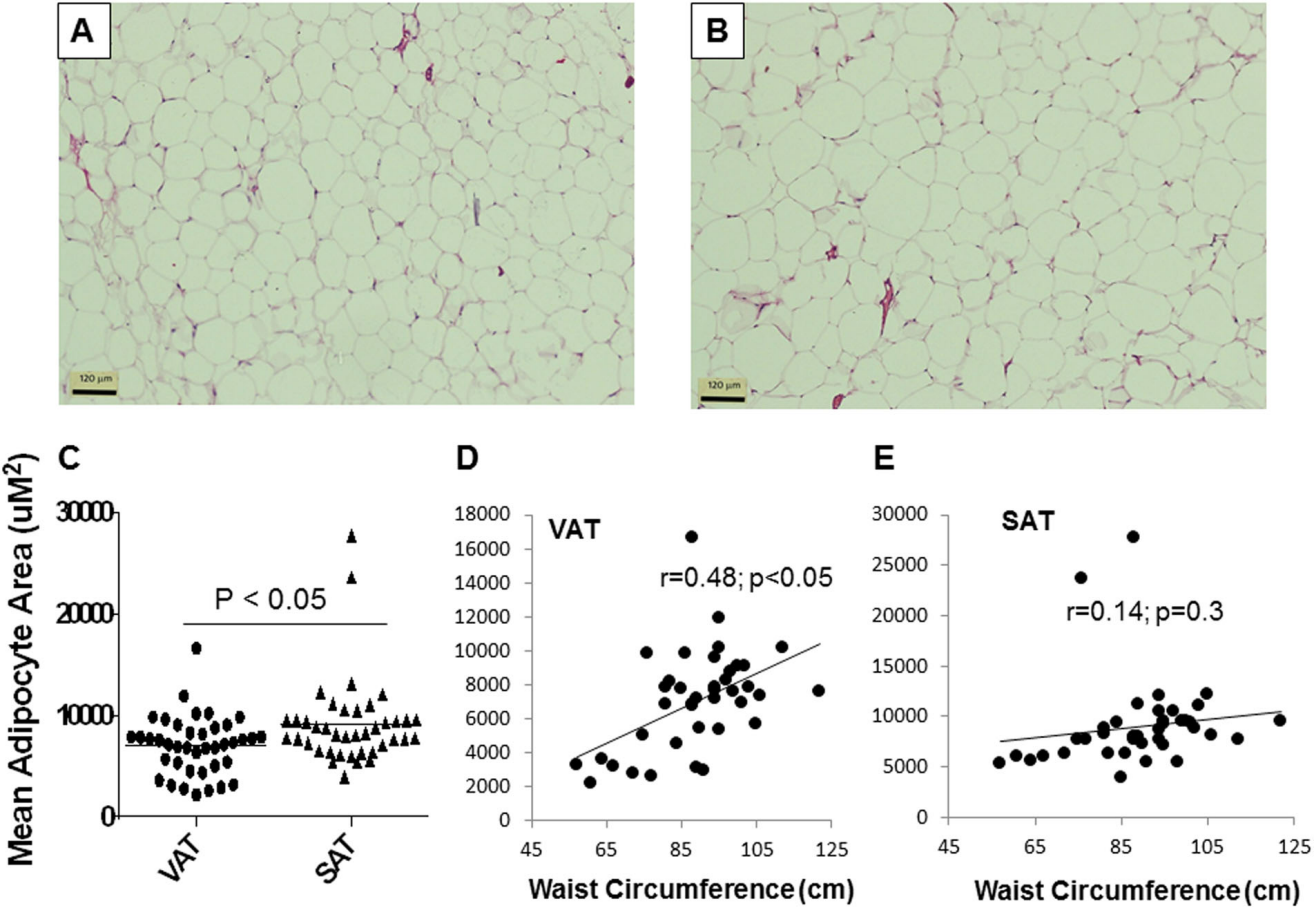
Depot-specific and adiposity-related changes in mean adipocyte area. Representative H&E stained sections of VAT (**a**) and SAT (**b**) are shown. Mean adipocyte area of VAT vs. SAT (**c**), correlations between mean adipocyte area and waist circumference in VAT (**d**) and SAT (**e**) are shown. *N* = 40. VAT: visceral adipose tissue, SAT: subcutaneous adipose tissue

Figure 5 shows that adipocytes were larger in the subcutaneous depot compared to the visceral depot. Adipocytes in the VAT became progressively larger when the BMI category increased. This relationship was less marked in the SAT. VAT mean adipocyte area significantly correlated with BMI, waist circumference and mean anterior abdominal wall thickness (Fig. 5), suggesting that visceral adipose tissue expands mainly by hypertrophy. In contrast, the mean adipocyte area of the SAT did not show correlations with BMI, waist circumference or anterior abdominal wall thickness, suggesting that this adipose tissue depot mainly expands via adipocyte hyperplasia rather than hypertrophy.

## Discussion

The aim of the current study was to investigate the early mechanistic derangements of glucose metabolism leading to type-2 diabetes (T2DM) in South Asian women. Therefore, we studied a sample of women with all categories of BMI and a spectrum of age. We identified increasing body mass, especially visceral adiposity, and aging as possible factors associated with insulin resistance in these women. Serum level of the adipocytokine resistin was significantly higher in women with IFG and positively correlated with waist circumference. Moreover, women with IFG had a higher percentage of resistin positive cells in adipose tissue. To our knowledge, this is the first study to perform resistin immunohistochemistry in human adipose tissue and show that adipose tissue resistin expression is associated with dysglycemia. Thus, it is plausible that both adipose tissue and systemic resistin are linked to dysglycemia in South Asian women.

### Factors associated with insulin resistance

The prevalence of T2DM has increased alarmingly in the recent past and South Asia has not been spared of this heavy burden^1^. Insulin resistance and a decreased insulin secretory capacity of the pancreas are the two key factors important in the pathogenesis of T2DM^16,17^. Therefore, identifying mechanisms responsible for insulin resistance is of interest. In the current study, we identified waist circumference and age as two factors associated with insulin resistance. These findings are in agreement with previous studies which have shown that both waist circumference^18^ and visceral adiposity^19^, as well as age^20^, are factors associated with insulin resistance and metabolic risk. While there was an association between HOMA-IR score and waist circumference, we did not find a significant association between BMI and HOMA-IR score. This indicates that body fat distribution, rather than body mass per se may be linked to insulin resistance in South Asian women.

There are several mechanisms which link increased visceral adiposity with insulin resistance^4,21^. First, a chronic low-grade inflammation occurring in adipose tissue characterized by immune cell infiltration is linked to insulin resistance^12,22^. Second, the dysregulated adipocytokine secretory patterns from adipose tissue leading to a pro-inflammatory state can lead to insulin resistance in insulin-sensitive tissues^12,23^. Third, a “lipid-spillover” effect leading to ectopic fat deposition in liver and skeletal muscle can impair insulin signaling and downstream metabolism culminating in insulin resistance^21,24^. To explore the aforementioned first mechanism, we examined H&E stained sections of VAT and SAT for crown-like structures and for the presence of macrophages by immunostaining. While we did find a correlation between the number of CLS per 25 fields of x10 objective with both BMI and anterior abdominal wall thickness, a significant number of CLS was found only at higher BMI levels. Moreover, there was no significant difference in the number of CLS between women with IFG vs. ones with NFG. In contrast, some studies, especially in morbidly obese Caucasian subjects, have shown that adipose tissue macrophages are associated with systemic insulin resistance^25^. Taken together, this suggests that adipose tissue immune cell infiltration is unlikely to be a major mechanism for dysglycemia in South Asian women.

To assess the endocrine function of adipose tissue, we measured the serum levels of several adipokines. We found that serum resistin was on average around 60% higher in women with IFG compared to those with NFG. Resistin is an adipokine secreted by adipocytes, which is increased in obesity and induces insulin resistance in rodents^13^. Moreover, resistin knockout exerts metabolic benefits in rodents^26^. However, the link between resistin, obesity and insulin resistance is inconsistent in humans, with some studies showing a positive association between serum resistin and insulin resistance^27,28^, while others report no such relationship^29,30^. According to findings from the current study, it is likely that resistin is a key link between visceral adiposity and dysglycemia in South Asian women.

### Resistin and insulin resistance

The source of serum resistin in humans is debatable. While resistin is considered to be an adipocytokine in rodents, resistin expression is highest in the bone marrow immune cells in humans^31^. While human adipocytes express both resistin gene and protein^32^, some studies suggest that macrophages may be the major source of resistin in adipose tissue^33^. In the current study, we performed immunohistochemistry to identify resistin protein expression in adipose tissue. Resistin expression in adipocytes was assessed and granular cytoplasmic positivity was regarded as true positivity. The amount of resistin positive adipocytes in a given tissue section was expressed as a percentage of all adipocytes by microscopic assessment. We did not use an automated method to quantify resistin because resistin positive cells were not uniformly distributed and there were many other non-specific peroxidase staining as found in plasma in blood vessels. This type of analysis is recommended when there are only few positive cells for an immunostain^34^. We found that women with IFG had a higher percentage of resistin positive cells, suggesting that local adipose tissue resistin may affect glucose homeostasis. Mechanistically, resistin induces insulin resistance by interfering with the insulin signaling cascade, via activation of the serine kinases Jun NH(2)-terminal kinase and p38 mitogen-activated protein kinase and via activating the Toll-like receptor 4^35^. Indeed, resistin inhibits glucose uptake by human adipocytes^36^ and rodent skeletal muscle cells^37,38^ in vitro. Since adipose tissue resistin secretion is not upregulated by lipopolysaccharide^39^, it cannot be considered as a marker of adipose tissue inflammation. Therefore, it is likely that resistin is an adipocytokine, which has local as well as systemic effects on glucose homeostasis.

While we found that both serum and adipose tissue resistin were higher in women with IFG, there was no correlation between the serum and adipose tissue levels of this adipokine. Therefore, it is unlikely that adipose tissue is the only source of resistin in these subjects. It is likely that in addition to immune cells, liver may also be a significant source of resistin in humans^39^.

### Inflammatory markers, adipocytokines, and dysglycemia

In the current study, we did not find any significant differences in serum adiponectin, leptin, TNF-alpha, MCP-1, IL-6, IL-8, IL-10, chemerin, or omentin-1 levels between women with NFG vs. those with IFG. Previous studies have reported that South Asians have lower serum adiponectin levels compared to Caucasians^40^, which has been pointed as a reason for the higher risk of T2DM among the former. Further, some studies have shown that adiponectin levels are inversely related to subsequent diabetes risk in Asians^41^. There are some reports of other cytokines, such as TNF-alpha^42^, IL-6, MCP-1 and chemerin being increased^12,30^, and omentin-1 decreased in obesity and metabolic syndrome^30^. However, we did not find such associations.

### Adipose tissue expansion in obesity

Adipocyte size is a strong determinant of the adipokine secretory patterns, with larger adipocytes producing more pro-inflammatory adipokines^43^. Although we found that there was a trend for a positive correlation between serum resistin and mean adipocyte area in VAT, this was not significant. Further, we did not find associations between adipocyte size and other serum adipokine levels. Previous studies have shown that South Asians have an increased visceral adipocyte area compared to Caucasians^40^. The differences in insulin and adiponectin between these two populations are attributed to this difference in adipocyte size^40^. Adipocyte hypertrophy is a maladaptive response to positive energy balance^44^. During positive energy balance, excess energy is stored in adipose tissue in the form of fat. The lipid storage capacity of the adipose tissue is increased by either adipocyte hyperplasia or hypertrophy. When there is a defect in adipose tissue expansion, especially in adipogenesis and adipocyte hyperplasia, adipocytes can undergo hypertrophy and ectopic fat deposition occurs in liver and skeletal muscle leading to insulin resistance^21,24^. In the current study, we found positive associations between VAT mean adipocyte area and all indices of adiposity (BMI, waist circumference, and anterior abdominal wall thickness), while no such association was found in SAT. This potentially indicates: (A) a better ability of SAT to expand without adipocyte hypertrophy, whereas VAT expands mainly by hypertrophy and (B) lipids being partitioned towards VAT, even with small increases in adiposity.

In conclusion, insulin resistance was associated with age and central adiposity in South Asian women. Serum and adipose tissue resistin may be an important adipocytokine linking central adiposity, insulin resistance and dysglycemia in these women.
